# Oral Methylphenidate Treatment of an Adolescent ADHD Rat Model Does Not Alter Cocaine-Conditioned Place Preference during Adulthood: A Negative Report

**DOI:** 10.20900/jpbs.20190021

**Published:** 2019-12-30

**Authors:** Yanli Zhang-James, David R. Lloyd, Michael L. James, Lina Yang, Jerry B. Richards, Stephen V. Faraone

**Affiliations:** 1 Department of Psychiatry and Behavioral Sciences, State University of New York Upstate Medical University, Syracuse, NY 13210, USA; 2 Department of Medicine, Jacobs School of Medicine and Biomedical Sciences, State University of New York at Buffalo, Buffalo NY 14203, USA; 3 Department of Cell and Developmental Biology, State University of New York Upstate Medical University, Syracuse, NY 13210, USA; 4 Department of Public Health Sciences, Penn State College of Medicine, Pennsylvania State University, Hershey, PA 17033, USA; 5 Department of Psychology, State University of New York at Buffalo Ringgold standard institution, 1021 Main Street, Buffalo, NY 14203, USA

**Keywords:** methylphenidate, ADHD, cocaine, conditioned place preference, locomotion

## Abstract

The stimulant, methylphenidate (MPH), is commonly used to treat attention deficit hyperactivity disorder (ADHD) and has been increasingly prescribed for school age children and adolescents. Concerns regarding its long-term effects on later substance use disorders (SUDs) have been raised. Previous animal studies have produced contradictory results regarding whether early exposure to MPH increases or protects against SUD in adulthood. The goal of our study was to determine if clinically relevant doses of MPH during adolescence alter cocaine responsiveness in adulthood in a rat model of ADHD, the spontaneous hypertensive rat (SHR). We pretreated SHRs with saline or MPH (2.5 mg/kg once or twice day) via oral gavage during their dark cycle from postnatal day 35 (p35) to p44. Adult rats (p80) were assessed in an eight-session cocaine-conditioned place preference test (CPP). Four doses of cocaine were administered via intraperitoneal injection (i.p.) during the conditioning sessions: 1, 5, 10 and 20 mg/kg. Once per day MPH treatment had a small sensitizing effect on baseline general locomotor activity in a novel environment at p80 as well as a limited suppressive effect on reward-specific locomotor activity as measured by the decreased preference to enter the cocaine-paired chamber. This treatment did not have any effect on the amount of time that rats chose to spend in the cocaine-paired chamber. Twice per day MPH treatment had no effect on locomotion or drug-preference. Our results suggest that MPH treatment of ADHD rats during adolescence does not alter preference for cocaine in adulthood.

## INTRODUCTION

The stimulant methylphenidate (MPH) treats attention deficit hyperactivity disorder (ADHD) [1] and is often prescribed for prolonged periods given that the disorder persists into adulthood in two-thirds of cases [2]. MPH increases extracellular dopamine by blocking the dopamine transporter (DAT). This mechanism underlies both its therapeutic and reinforcing effects [3]. MPH is abused by adolescents and adults [4–6] and the diversion and misuse of ADHD medications is a clinical concern [7,8]. Adolescent rats readily self-administer MPH, as they do cocaine [9]. Although a slow uptake of medication in the brain via oral treatment has a lower potential for abuse or diversion [10], it is still not clear whether long-term exposure to therapeutic dose of MPH during youth alters the risk for substance abuse in adulthood.

A meta-analysis suggested a protective effect of early methylphenidate treatment on the development of SUDs in adolescence, but not adulthood [11]. Since then, several studies reported either no association of SUDs with early stimulant treatment [12–15], or a protective effect [16–19]. A randomized, placebo-controlled trial found that stimulant treatment improved SUDs outcomes in adolescents with co-morbid attention and conduct problems [20,21]. This work reported that age at stimulant treatment initiation was positively related to the later development of non-alcohol SUDs. Groenman *et al.* [22] also reported that children who started stimulant medication at younger ages were better protected against later SUDs. But in that study, the effect of age of first stimulant use on SUD development diminished with age.

Animal studies have produced far more inconsistent results. Some found that MPH treatment increased aversion or decreased liking to cocaine [23–26]. Others found that MPH treatment led to enhanced cocaine self-administration, ethanol intake, behavioral sensitization, or drug-conditioned place preference [27–30]. These studies differed in many methodological features including: dose, route of administration, age, gender, individual genetic predisposition (animal strains) and time of treatment (during dark or light cycles of the rodent circadian rhythm), that contributed to variation in the data [31,32]. A typical treatment dose of MPH for ADHD administered orally (e.g., b.i.d, t.i.d. or slow-release) achieves an 8–40 ng/mL plasma peak level after approximately 2 h in humans [32–34]. This induces a slow-steady increase of brain dopamine [4,32,35]. A 1–3 mg/kg oral dose in rats was estimated to produce a similar plasma peak level but with a much shorter half-life due to the between species differences in drug absorption and metabolism [32,36]. Unfortunately, most rat studies used i.p. or intravenous injections of this or higher dose levels, leading to faster and larger increases of plasma drug levels and brain dopamine levels, which are often associated with locomotor stimulation and sensitization instead of a calming effect for hyperactivity [37–39]. Gaytan *et al.* [40] have found that the most robust sensitization occurred if treatment was during the light phase, while no sensitization was observed if treatment was during the dark phase. Treatment given during the dark cycle was also associated with smaller effects of high dose MPH on stereotypic behaviors [41].

Rat strains also play important roles in different response to MPH treatment [42,43]. For example, repeated 2.5 mg/kg MPH treatment elicited locomotor sensitization in behaviorally “normal” control strains, such as Sprague Dawley (SD) and Wistar Kyoto (WKY) rats, but not in the SHR rat, a validated rat model for ADHD [43]. Similar to ADHD human patients, SHR rats could benefit from a paradoxical calming effect of stimulant treatment, whereas “normal” individuals and rats experienced locomotor activation [36,44,45]. SHR rats readily self-administered MPH and developed conditioned place preference to MPH confirming a rewarding effect of MPH for SHR rats. However, SHR rats learned MPH self-administration faster and responded more to MPH infusions than did Wistar rats, particularly more so in the adolescent SHR rats [46,47]. This is in concordance with higher rates of SUDs in ADHD than non-ADHD youth [14].

Among 400 articles retrieved from PubMed by searching “methylphenidate AND cocaine” or “methylphenidate AND drug abuse” in non-human species, 238 used rat species. However, only 10 studies used SHR rats. Five of these examined the effects of MPH treatment during adolescence on adult animals’ drug response or use behavior [26,30,46,48,49]. Among all 10 studies, only one specified that the experiments were conducted during the rats’ dark cycle [48]. Although all the treatment studies modeled clinically relevant doses, the majority used i.p. injections; only two administered MPH orally [30,48]. Soeters *et al*. [48] was the only study that treated SHR rats with 2 mg/kg MPH via oral consumption in their dark cycle. The authors did not find any effect of MPH treatment during adolescence (p21–35) on ethanol consumption later in adulthood, although they found that SHRs consumed less ethanol voluntarily than did the control WKY rats, which is contrary to the clinical observation of a higher risk of alcohol abuse in ADHD individuals [22].

To address the limitations of prior animal studies of the link between MPH treatment and subsequent substance use behaviors, our study treated adolescent SHR rats orally during the dark cycle and examined their cocaine-conditioned place preference behavior during adulthood. To compensate for the shorter half-life of MPH in rats, we also compared the effects of once per day and twice per day treatments.

## METHODS

### Animals

108 male SHR rats (4 weeks old) obtained from Charles River Laboratories were housed in pairs in plastic cages (24 cm × 46 cm × 20 cm) with *ad libitum* access to food and water and kept on a 12 h reverse light cycle (lights on from 6 pm to 6 am). All drug treatment and behavioral test procedures were approved by the Animal Care and Use Committee of the State University of New York at Buffalo.

### Conditioning Apparatus

Eight in-house constructed conditioning apparatuses were used. Each apparatus had three compartments separated by removable dividers. The left and right compartments were 23 cm × 35 cm and featured differential tactile and visual cues. Tactile cues consisted of stainless-steel square wire-mesh grid flooring with either 0.8 cm or 1.5 cm grid-spacing. Visual cues consisted of two-colored walls which were either black or white. The tactile and visual cues were uncorrelated and evenly distributed between left and right compartments. The middle compartment was 16.5 cm × 15 cm and when the compartment dividers were removed had two openings measuring 15.9 cm × 11.7 cm that allowed subjects to freely travel between compartments. All compartments were 29 cm tall and were covered using a removable Plexiglas top.

### Drugs

Animals were pretreated with (±)-methylphenidate hydrochloride (MPH) by oral gavage on days p35–44 twice a day at 9 am and 1 pm. MPH was dissolved in saline (SAL) at a concentration of 2.5 mg/mL and administered to animals at 2.5 mg/kg so that rats received equal gavage volumes by weight. Animals were randomly divided into three pretreatment groups. The control group received SAL twice a day. MPH was given either once per day (MPH1 group, MPH gavage at 9 am and SAL at 1 pm) or twice per day (MPH2 group, MPH gavage at both times).

(−)-Cocaine hydrochloride (gifted by NIDA-RIT Log no: 13070–12C, ref. #013277) was dissolved in saline and injected (intraperitoneal) to rats from p82 to p86 during CPP conditioning. Only rats that were weighted between 220 g and 335 g (*M* = 282.0 g, *SD* = 19.7 g) at the time of testing (total = 89) were used for conditioned place preference (CPP) testing. Animals from each pretreatment group were randomly assigned to four different cocaine dose groups (1, 5, 10 and 20 mg/kg) and received injections of equal volumes by weight.

### Test for Cocaine-Induced Conditioned Preference

CPP testing used an unbiased procedure and consisted of three phases: pre-conditioning, conditioning and post-conditioning. Sessions were 30 min in duration. During the pre-conditioning session (p81), all dividers were removed, and animals were placed in the center compartment and allowed to freely travel between compartments. The conditioning phase consisted of test sessions 2–7. Immediately prior to conditioning sessions 2, 4, and 6, half the rats were injected with one of four doses of cocaine (COC) and confined in the paired cocaine compartments while the other half were injected with saline and placed in the paired saline compartments. During the conditioning phase the dividers were in place and animals could not travel between compartments. Immediately prior to conditioning sessions 3, 5, and 7, rats that were previously injected with cocaine were injected with saline and placed in the paired saline compartment and those rats that were previously injected with saline were injected with cocaine and placed in the paired cocaine compartment. Animals received a total of 3 saline and 3 cocaine conditioning tests. The scores from the pre-conditioning phase were used to determine the unconditioned compartment preference. Because a biased design, *i.e.*, paring cocaine with the non-preferred compartment, is susceptible for false positive findings [50], we randomly assigned half of the animals to receive drug in their preferred compartment and the other half to receive drug in their non-preferred compartment during the conditioning phase. The post-conditioning phase consisted of one session. Animals were placed in the center compartment, with all dividers removed so that they were able to freely travel between compartments. Both pre- and post-conditioning phases were video-taped for analysis.

### Data Analysis

The videos were analyzed using ANY-maze software by an experimenter blinded to the treatment conditions. The software tracks animals’ heads and defines the rats to be in a given compartment if ≥85% of its body was in that compartment. Three additional animals were excluded from the analysis because software failed to correctly track the animals. The final number of animals used for the analysis was 86 with group sizes ranging from 6 to 9. The primary measurements were the number of entries, the distance traveled, and amount of time spent in each compartment. Previous studies of cocaine CPP have primarily examined the time spent in the drug-paired chambers. We sought to examine a full spectrum of behavioral measurements that are available in our data in order to disentangle the reinforcing effect of the drug from the animals’ locomotor sensitization and hyperactivity. A total recording time of 30 min for each rat was divided into six 5-minute segments. 5-Minute segments were chosen based on others and our previous studies to examine locomotor activity and habituation in experimental chambers [51,52]. Only the first 5 segments were used because some of the videos were truncated during the last segment. For the pre-conditioning and post-conditioning phases, general locomotion was represented by the sum of the number of entries or distance traveled in all three compartments. The preference to cocaine was represented by subtracting the number of entries, distance traveled, and time spent in the cocaine-paired compartment in the pre-conditioning session from those of the post-conditioning session. We also calculated the relative preference for cocaine *vs* saline by subtracting the above measurements of the saline-paired compartment from those of the cocaine-paired compartment during only the post-conditioning sessions. We examined all the measurements in a longitudinal data format consisting of five 5-minute segments and used a random effect general linear regression model to evaluate the effects of MPH pretreatment and cocaine dose in STATA 12.0.

## RESULTS

### General Locomotor Activity

**Figure 1.**
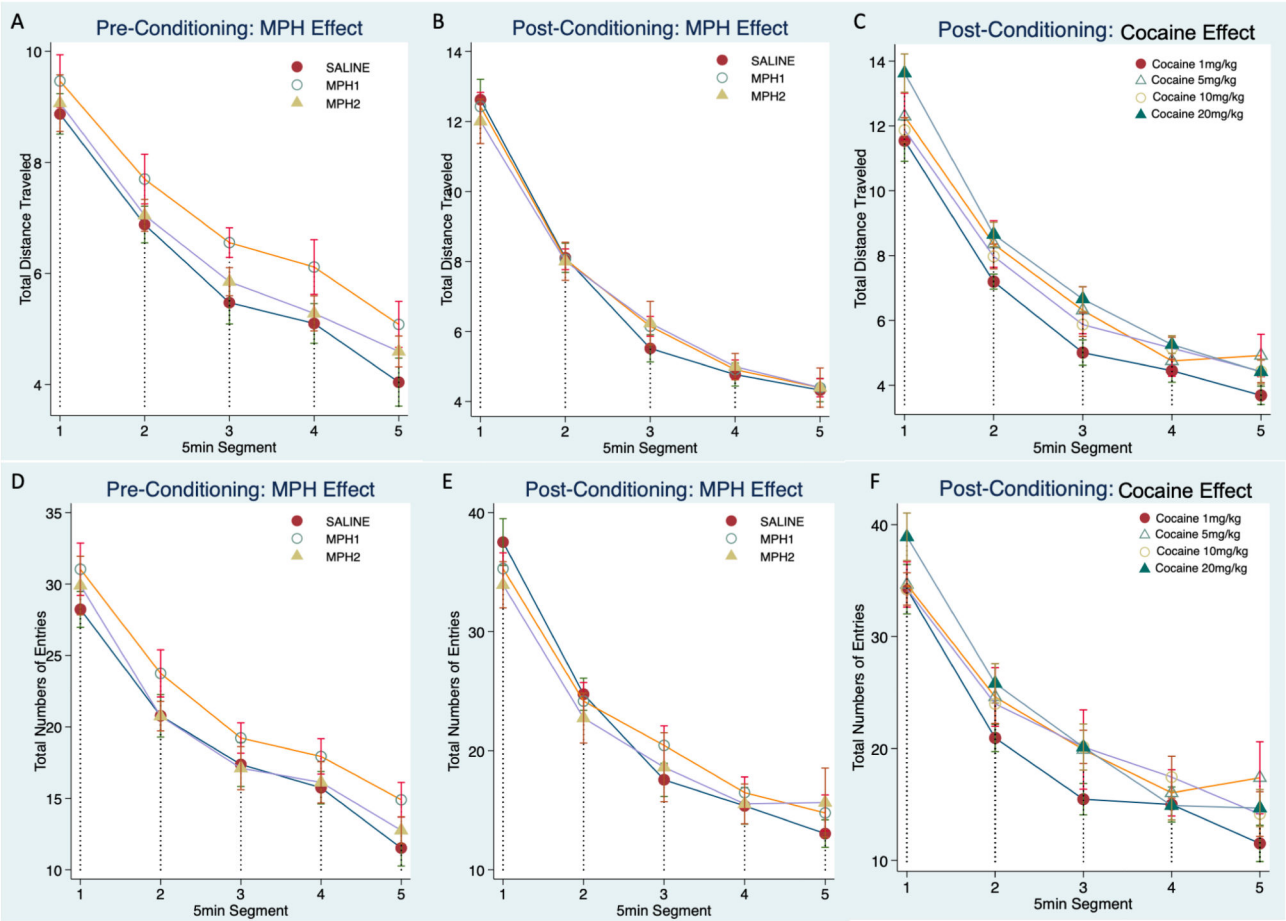
General locomotor activity during the pre- and post-conditioning tests. The total distance traveled (**A**–**C**) and the total numbers of entries (**D**–**F**) to all three compartments were plotted over five segments to show the general locomotor and habituation. Group mean and standard errors were shown for each time segment. Animals were grouped in individual figures to show the main effect of MPH pretreatment for the pre-conditioning test (**A**,**D**) and post-conditioning test(**B**,**E**), and the main effect of the cocaine doses (**C**,**F**) at the post-conditioning test. MPH1, 2.5 mg/kg MPH once per day treatment; MPH2, 2.5 mg/kg MPH twice per day treatment.

We assessed the general locomotor activity during the pre- and post-conditioning sessions. Figure 1A,D plots total number of entries and distance traveled over five 5-minute segments to show the main effect of MPH pre-treatment during the pre-conditioning phase, which assessed the baseline activity of the pre-treated animals in adulthood. Locomotor activity during the post-conditioning phase were plotted for the main effects of MPH-pretreatment (Figure 1B,E) and the main effects of cocaine doses (Figure 1C,F). The main effects of time segments were significant for both the total number of entries during pre-conditioning (χ^2^_(4)_ = 400.75, *p* < 0.0001) and post-conditioning sessions (χ^2^_(4)_ = 324.89, *p* < 0.0001), and the total distance traveled during pre-conditioning (χ^2^_(4)_ = 520.63, *p* < 0.0001) and post-conditioning sessions (χ^2^_(4)_ =1650.08, *p* < 0.0001).

MPH pretreatment had no effect on the locomotor activity. The small increase of total distance traveled observed for the MPH1 group compared with the SAL group during the pre-conditioning phase was non-significant (χ^2^_(2)_ = 4.84, *p* = 0.09, Figure 1A). The MPH2 group did not differ from the SAL group. A similar pattern was observed for the total entries but was also not significant (Figure 1D). The randomly assigned cocaine treatment groups were not different during the pre-conditioning phase (not shown).

During the post-conditioning phase, we also found no effects of MPH pretreatment, nor the interaction of MPH and time-segment (Figure 1B,E). There was a significant effect of interaction between cocaine dose and time-segment (χ^2^_(15)_ = 39.21, *p* = 0.0006) for the total entries, primarily due to the lower number of entries of the 1 mg/kg cocaine dose group during time segments 2, 3 and 5 (Figure 1F). The overall effect of cocaine dose on the total distance traveled was modest, and there was no effect of interaction between cocaine dose and time-segment.

**Figure 2.**
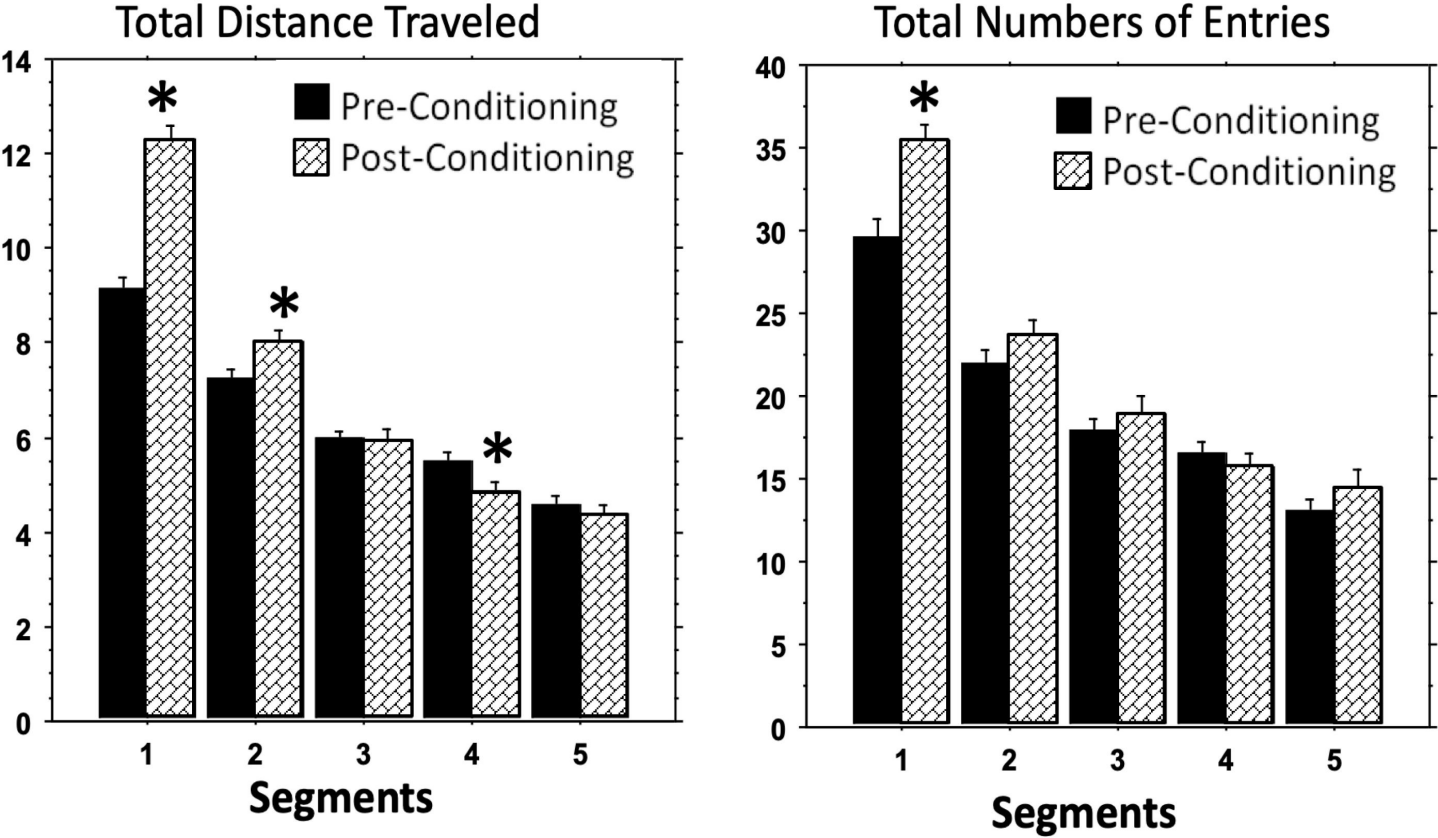
Cocaine conditioning induced locomotion stimulation. The total distance traveled and the total numbers of entries to all three compartments were plotted for all groups combined to show the main effect of the cocaine conditioning induced locomotion stimulation over time. * indicated significant difference (*p* < 0.05) between pre- and post-conditioning tests.

Cocaine conditioning significantly increased the spontaneous locomotor activity in the test apparatus, particularly during the first 10 min as measured by total distance traveled (first 5-minute segment: *F*_(1,173)_ = 64.42, *p* < 0.0001; second 5-minute segment: *F*_(1,171)_ = 6.01, *p* = 0.015) and during the first 5minutes as measured by total number of entries (*F*_(1,176)_ = 16.74, *p* = 0.0001) (Figures 1 and 2). The effect of conditioning-induced locomotor stimulation disappeared after the first 10 min as measured by distance traveled and after 5 minutes as measured by total number of entries. There was a moderate effect of locomotor depression during the fourth 5 minute segment as measured by total distance traveled (*F*_(1,173)_ = 4.45, *p* = 0.034), but that effect disappeared during the final segment (Figure 2 Left). Such time-dependent change of activity pattern was similarly observed for all cocaine and MPH treatment groups, and there was no effect of MPH or cocaine dose on the conditioning-induced locomotor increase.

### Effects of MPH Pretreatment on Cocaine-Conditioned Place Preference

**Figure 3.**
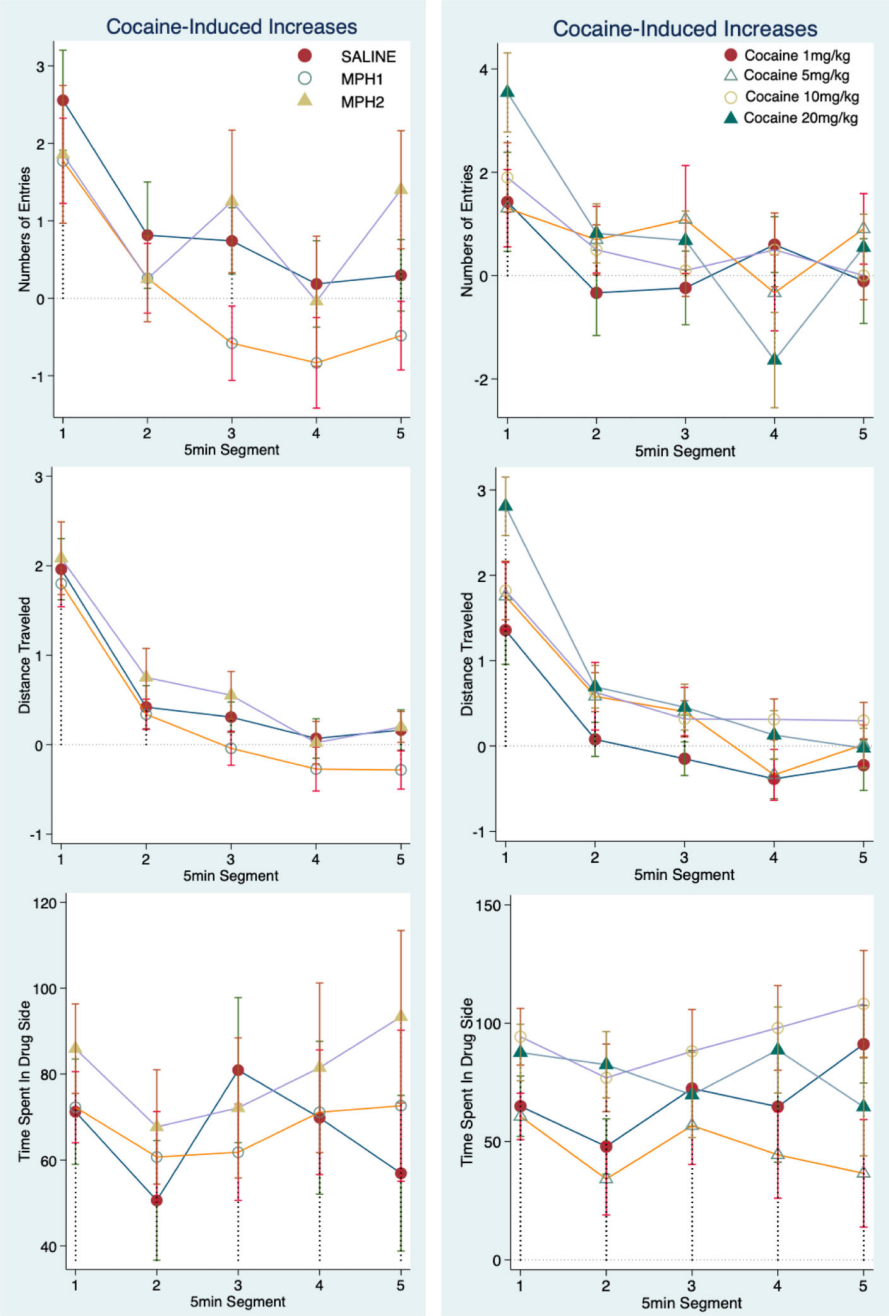
Cocaine-conditioned place preference measures. Cocaine preference was calculated by the post-conditioning increase of all three measures to the drug-paired compartment from those of the pre-conditioning baselines: the number of entries (Top Row), the distance traveled (Middle Row) and the time spent in the cocaine-paired compartment (Bottom Row). The group means and standard errors were plotted over time segments to show the effects of MPH pretreatment groups (Left Panel) and cocaine doses (Right Panel). MPH1, 2.5 mg/kg MPH once per day treatment; MPH2, 2.5 mg/kg MPH twice per day treatment.

Preference for cocaine was calculated as the post- and pre-conditioning difference (cocaine-conditioning induced increase) for all three measurements: number of entries to the cocaine-paired compartment, distance traveled, and time spent in the cocaine-paired compartment. Figure 3 plots these measurements to show the main effect of MPH pretreatment (Left) and cocaine dose (Right) over time. There was a significant effect of time segment on the numbers of entries (χ^2^_(4)_ = 34.29, *p* < 0.0001) and distance traveled (*F*_(4,427)_ = 32.63, *p* < 0.0001), but not for time spent in the drug-paired compartment. Cocaine-induced increases of locomotor activity (entries and distance traveled) within the cocaine-paired compartment were the most robust in the first 5-minute segment and subsequently decreased drastically afterwards to the levels of the pre-conditioning phase (Figure 3 Top two rows).

We found a modest but overall non-significant effect of MPH pretreatment on cocaine–induced increase in number of entries to the drug-paired compartment (χ^2^_(4)_ = 4.88, *p* = 0.087; Figure 3 Top Left). The effect was due to reduced increase of entries to the drug-paired compartment for the MPH1 treatment group in comparison with the saline treatment (χ^2^_(4)_ = 5.87, *p* = 0.015). This suppression was observed only during the last three 5-minute segments, after cocaine-induced locomotion stimulation had waned. MPH2 treatment had no effect on this measure. There was no effect of cocaine dose or interaction of cocaine dose, MPH pretreatment and time on the increased entries (Figure 3 Top row). We also found a modest but significant effect of cocaine dose effect on the increase of distance traveled in the cocaine-paired compartment (Figure 3 Middle Right, χ^2^_(3)_ = 8.4, *p* = 0.039), due to lower increase for the 1 mg/kg cocaine group. There was no effect of either MPH pretreatment, time segments or cocaine dose on the increase of time spent in the cocaine-paired compartment (Figure 3 Bottom row).

**Figure 4.**
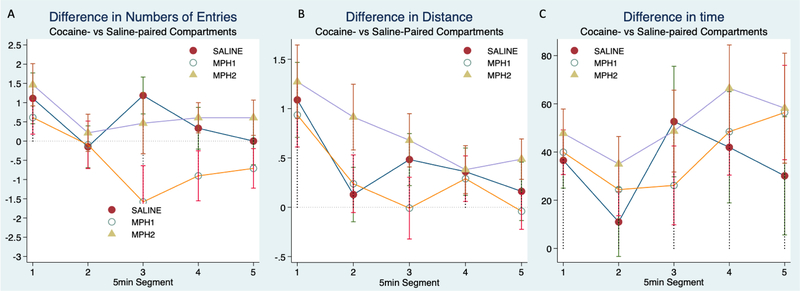
A relative preference to the cocaine- vs saline-paired compartments was calculated by subtracting the number of entries (**A**), distance traveled (**B**), or time spent (**C**) in the saline compartment from that of the cocaine-paired compartment during the post-conditioning phase. This relative preference was plotted over time segment to show the main effect of MPH treatment groups.

**Figure 5.**
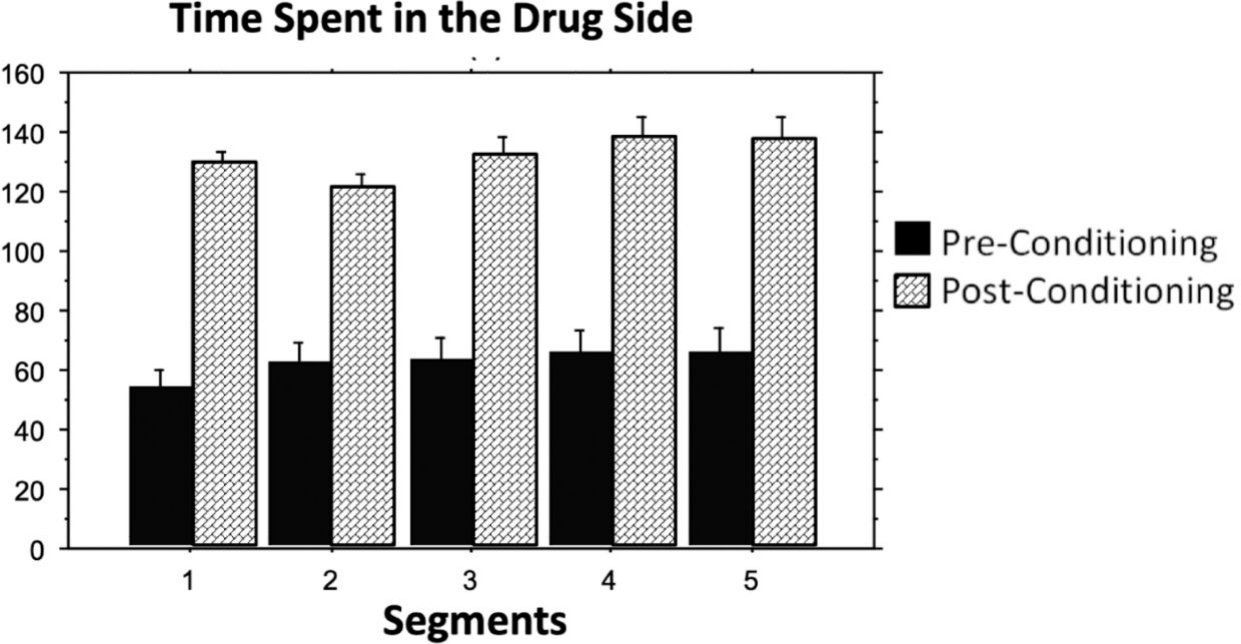
The time animals spent in the cocaine-paired compartment was plotted for pre- and post-conditioning phases to show the effectiveness of cocaine conditioning and the lack of change over time.

We also calculated a relative preference to the cocaine-paired compartment by subtracting the measurements to the saline-paired compartment from that of the cocaine-paired compartment during only the post-conditioning session (Figure 4). Because half of the animals were paired with cocaine in their preferred compartments and half in their non-preferred compartments, we included it as a covariate in our model. Overall, we found that the cocaine induced changes in either preferred or non-preferred compartments were not significantly different and that the effect of cocaine dose was also not significant. Consistent with what we have seen, time segment effect was significant for both the numbers of entries (Figure 4A, χ^2^_(4)_ = 10.45, *p* = 0.033), and distance traveled (Figure 4B, χ^2^_(4)_ = 30.52, *p* < 0.0001), but not for the time (Figure 4C). The lack of time segment effect on the increased time spent in the cocaine-paired compartment was further shown in Figure 5. Figure 4 showed the effect of MPH pretreatment on these measurements. The significant effect of MPH treatment on the differences in numbers of entries remained (χ^2^_(2)_ = 6.22, *p* = 0.04, Figure 4A), with MPH1 pretreatment showing fewer entries to the cocaine-paired compartments than to the saline-paired compartments. Because an increase of entries to the drug-paired chambers could simply be due to enhanced locomotor stimulation, we included the total number of entries to all chambers at the post-conditioning phase as a covariate in the analysis. We found that the total number of entries had no effect on this measure of relative preference to cocaine. The addition of this covariate completely eliminated the effect of time segments, confirming that the total number of entries represents motor habituation. The effect of MPH pretreatment, namely the suppressive effect of MPH1 treatment on the difference in entries, however, remained significant (χ^2^_(2)_ = 6.34, *p* = 0.04).

## DISCUSSION

Animal models are widely used in studies of childhood and adolescent MPH treatment effects on adult substance abuse behaviors. However, the majority of these studies have significant methodological problems that have led to contradictory results and further obfuscated the issue. Addressing this issue in a manner that is relevant to the clinical treatment of ADHD requires selecting the proper MPH dose and the route and timing of drug administration. We used SHR rats, the most widely used and validated genetic rat model for ADHD. Our goal was to address the issues that limited the interpretation of previous studies and to determine if the clinically relevant use of MPH in an adolescent ADHD rat model would lead to changes in their cocaine-conditioned place preference behavior in adulthood.

All four doses of cocaine induced significant locomotion stimulation and increased preference for the cocaine-paired compartment. The observation that cocaine is rewarding for the SHR is consistent with previous work [26,30]. However, one study did not observe a rewarding effect at the 1 mg/kg dose [26].This discrepancy could be because that our experiment conducted three trials of cocaine and saline paring (6 days total), while Augusyniak *et al.* only conducted two trials of cocaine and saline pairing (4 days total) [26]. The differences could also be due to the treatment regimen, (light vs. dark cycle) and genetic variations in the SHR strains from different vendors [40,53]. It is not surprising to see the lack of dose-response curves in the CPP results. Augusyniak *et al.* also showed similar levels of preference to the cocaine chambers for all effective doses (5, 10 and 20 mg/kg) in SHR rats [26]. We did observe a small but significant cocaine dose-dependent locomotor stimulation (Figure 1C). Self-administration paradigm maybe better suited than the CPP paradigm for study of dose-dependent rewarding effects of cocaine.

One strength of our current study is detailed analysis of the CPP behavior during a prolonged period of time (25 min total), which revealed two separate behavioral dimensions in SHR rats: locomotor sensitization and conditioned preference to the cocaine. Locomotor sensitization was only present during the first 10 min of post-conditioning testing. Context alone was enough to elicit the behavioral sensitization for all the effective doses of cocaine used, and it did not require a challenge injection. In contrast, conditioned preference to cocaine, measured by the increased time that animals spent in the cocaine-paired compartment, was not time dependent. Once conditioning was well established, animals continued to spend more time in the drug-paired compartment, regardless of the decreasing number of entries into that chamber due to locomotion habituation. It is important to note that animal entries to the drug-specific compartment may reflect both the general locomotor stimulation and reward-invoked drug seeking. In our analysis of the number of entries to the drug vs. saline paired compartments, we statistically controlled the effects of general locomotor activation on the reward-specific increase of entries to the cocaine compartment. Interestingly, when general locomotion was taken into account, the specific change of entries to the cocaine compartment was no longer dependent on time. This observation was consistent with the lack of time segment effect on the cocaine preference measured by the time. Both of which support the conclusion that established cocaine conditioned preference does not change over time during the course of our CPP testing, although locomotion habituates over time.

Our main finding is the lack of effect of MPH pretreatments on the increased preference to cocaine-paired chambers as measured by the time. Five prior studies used SHR rats to examine the effect of MPH treatment in adolescence on drug response in adulthood. Three studies administered MPH once per day. Augustyniak *et al*. [26] found a reduced CPP to cocaine and De la Pena *et al.* [46] found a reduced CPP to methylphenidate. Both studies administered MPH via i.p. during the rats’ light cycles. Harvey *et al*. [30] used oral administration during the light cycle and found that it enhanced the SHR’s speed to acquire cocaine self-administration and exerted the opposite effect for Wistar rats. Two studies used a twice per day treatment regimen, one via i.p during the light cycle [29] and one via oral consumption during the dark cycle [48]. Both studies found no effect on adult male SHR rats’ voluntary alcohol consumption. Our study is the only one that examined both once and twice per day regimens via oral administration during rats’ dark cycles. Our results showed that neither treatment regimens had significant impact on animals’ preference to cocaine-paired chambers as measured by the time.

However, we did find a suppressive effect of MPH1 treatment on the number of entries to the cocaine-paired compartment. Interestingly this suppressive effect was only observed after the cocaine-associated locomotion stimulation had waned and the effect remained significant after removing the influence of general locomotion on the entries. The decrease of entries to drug side in the MPH1 treatment group may be due to reduced unnecessary and impulsive entries that rats made, considering that the total increased time that rats stayed in the drug side remained same for all pretreatment groups. A recent meta-analysis of SHR behavior did find a significant effect of MPH treatment on reducing impulsivity [54]. It is not clear why the effect on the entries was limited to the once per day treatment group. Considering that MPH has a much shorter half-life in rodents than in humans, a twice per day treatment paradigm likely offers a more consistent therapeutic plasma drug level over a longer duration. This treatment paradigm is more similar to human clinical use.

The main limitations of the study are small sample sizes and a balanced design of biased apparatus. Our final sample sizes were 6–9 animals per treatment group, which may have limited our ability to detect small changes. The sample size also prevented us from adopting a truly unbiased design. Future studies with more animals per group in unbiased apparatus will be needed to replicate our findings. Limitations in translational utility of inferring SUDs in human ADHD patients should be considered for several reasons. First, the negative findings in CPP test can only suggest a lack of change in sensitivity to the conditioned rewarding effect of cocaine [50]. Self-administration paradigm will be needed to directly address the link with addiction. Secondly, there are limitations associated with using SHR rats as the ADHD model. The adolescent SHR rats are the most widely used and validated animal model for ADHD. However, adult rats develop hypertension and it is not clear how hypertension affects the cocaine CPP behavior in adult rats. Furthermore, future studies with inclusion of and comparison with appropriate control strains are warranted to demonstrate the therapeutic effects of MPH for ADHD without any abusive liabilities of cocaine. Finally, ADHD is known to have high co-morbidity with mood disorders such as major depression and anxiety. Treatment with MPH and other stimulants has been shown to decrease the incidence and improve the symptoms of these disorders, both of which are known to independently contribute as risk factors to SUDs. Furthermore, improvement in key symptom areas such as impulsivity and subsequent improvements in social and occupational performance may profoundly influence outcomes for developing SUDs. The connection between impulsivity and drug seeking and using behaviors are well established and poor social and occupational outcomes contribute to low self-esteem, which itself is linked to SUDs. From the social, occupation and emotional perspective, rat studies of ADHD models have limited utility in explaining the potential protective effect of MPH treatment on subsequent SUDs observed in some clinical studies.
